# Synthetic Breast Ultrasound Images: A Study to Overcome Medical Data Sharing Barriers

**DOI:** 10.34133/research.0532

**Published:** 2024-12-03

**Authors:** JiaLe Xu, Qing Hua, XiaoHong Jia, YuHang Zheng, Qiao Hu, BaoYan Bai, Juan Miao, LiSha Zhu, MeiXiang Zhang, RuoLin Tao, YuHeng Li, Ting Luo, Jun Xie, XueBin Zheng, PengChen Gu, FengYuan Xing, Chuan He, YanYan Song, YiJie Dong, ShuJun Xia, JianQiao Zhou

**Affiliations:** ^1^ Department of Ultrasound, Ruijin Hospital, Shanghai Jiao Tong University School of Medicine, 200025 Shanghai, China.; ^2^ College of Health Science and Technology, Shanghai Jiao Tong University School of Medicine, 200025 Shanghai, China.; ^3^ Department of Ultrasound, The People’s Hospital of Guangxi Zhuang Autonomous Region, Nanning, 530021 Guangxi, China.; ^4^ Department of Ultrasound, Yan’an University Affiliated Hospital, Yan’an, 716000 Shaanxi, China.; ^5^ Department of Ultrasound, Zigong Fourth People’s Hospital, Zigong, 643000 Sichuan, China.; ^6^ Department of Ultrasound, Yichun City People’s Hospital, Yichun, 336000 Jiangxi, China.; ^7^ Shanghai Aitrox Technology Corporation Limited, 200050 Shanghai, China.; ^8^ Department of Biostatistics, Institute of Medical Sciences, Shanghai Jiao Tong University School of Medicine, 200025 Shanghai, China.

## Abstract

The vast potential of medical big data to enhance healthcare outcomes remains underutilized due to privacy concerns, which restrict cross-center data sharing and the construction of diverse, large-scale datasets. To address this challenge, we developed a deep generative model aimed at synthesizing medical data to overcome data sharing barriers, with a focus on breast ultrasound (US) image synthesis. Specifically, we introduce CoLDiT, a conditional latent diffusion model with a transformer backbone, to generate US images of breast lesions across various Breast Imaging Reporting and Data System (BI-RADS) categories. Using a training dataset of 9,705 US images from 5,243 patients across 202 hospitals with diverse US systems, CoLDiT generated breast US images without duplicating private information, as confirmed through nearest-neighbor analysis. Blinded reader studies further validated the realism of these images, with area under the receiver operating characteristic curve (AUC) scores ranging from 0.53 to 0.77. Additionally, synthetic breast US images effectively augmented the training dataset for BI-RADS classification, achieving performance comparable to that using an equal-sized training set comprising solely real images (*P* = 0.81 for AUC). Our findings suggest that synthetic data, such as CoLDiT-generated images, offer a viable, privacy-preserving solution to facilitate secure medical data sharing and advance the utilization of medical big data.

## Introduction

Medical big data, derived from diverse sources such as electronic health records, medical imaging, and biomarker profiles, are fundamentally reshaping the healthcare landscape by enhancing clinical decision-making and advancing medical research [1]. The use of artificial intelligence algorithms, particularly deep learning (DL) methods, has become increasingly prevalent in the analysis of medical big data to identify disease patterns, predict clinical events, provide tailored recommendations, and support resource allocation decisions [2–4]. Despite their potential, medical big data present substantial challenges, particularly regarding patient privacy [5]. The cross-center sharing of medical data, essential for constructing large and diverse datasets, raises privacy concerns and the risk of personal information misuse. Consequently, there has been substantial work on deidentification and differential privacy [6,7]. However, deidentification is prone to reidentification risks, and differential privacy often compromises data utility by introducing noise [8,9]. In regions with strict data sharing regulations, federated learning has been proposed as a potential solution, enabling collaborative model training without sharing raw data [10,11]. However, it remains vulnerable to privacy leakage from the model updates or the final model [12].

A promising alternative to address data sharing challenges is synthesizing medical data that preserve the statistical distribution of the original dataset while safeguarding patient privacy. Although medical data are diverse, this work focuses specifically on medical imaging data. Deep generative models, such as generative adversarial networks [13], variational autoencoders (VAE) [14], and diffusion models (DMs) [15], have demonstrated substantial potential for generating imaging data. Among these, DMs have recently achieved state-of-the-art performance in natural image synthesis [16], holding promise for medical imaging applications. However, DMs are resource-intensive, with slow sampling speeds and high computational demands. Innovations like the denoising diffusion implicit model have accelerated sampling [17], while latent diffusion models (LDMs) improve computational efficiency by operating in the latent space [18]. Conditional DMs have also benefited from classifier-free guidance, further enhancing sample quality [19]. Traditionally, DMs have employed convolutional U-Net architectures [15,20], but recent advances have explored the use of vision transformers (ViTs) as alternative backbones [21–23]. Diffusion transformers (DiTs), which integrate ViTs into LDMs, have demonstrated superior scalability and outperformed their U-Net counterparts on class-conditional ImageNet benchmarks [23]. Despite these advancements, their application in medical image synthesis remains underexplored. Motivated by this gap, we applied DiT-based LDMs to the synthesis of medical images, focusing on breast ultrasound (US) image synthesis as an initial exploration.

**Fig. 1.**
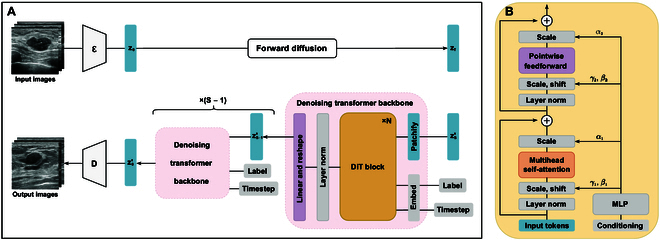
Schematic diagram of CoLDiT. (A) Input images are compressed using encoder ℇ to get $z_{0}$. The forward diffusion involves *T* steps of adding noise to turn $z_{0}$ into $z_{T}$. During the reverse diffusion, the denoising transformer backbone predicts the added noise and covariance of the noisy latent representatives, obtaining $z_{0}^{\prime }$ after *S* inference steps. Finally, $z_{0}^{\prime }$ is decoded by D to output synthetic images. (B) Each diffusion transformer (DiT) block follows the structure of the adaptive layer norm-zero (adaLN-Zero) block, receiving input tokens and conditioning embeddings, including Breast Imaging Reporting and Data System (BI-RADS) labels and noise timesteps. MLP, multilayer perceptron.

Here, we present CoLDiT, a conditional LDM with a DiT backbone, to generate high-resolution breast US images conditioned on Breast Imaging Reporting and Data System (BI-RADS) categories (BI-RADS 3, 4A, 4B, 4C, and 5) (Fig. 1). To our knowledge, this study represents the first investigation into the application of DiT-based LDMs in synthesizing US images. We evaluated the synthetic images regarding realism and privacy protection through 3 reader studies, 2 quantitative metrics—inception score (IS) and Fréchet inception distance (FID)—and a privacy evaluation test. Additionally, to evaluate the utility of CoLDiT in data augmentation, we conducted BI-RADS classification tasks using both real images and a combination of real and synthetic images as training data. Our findings demonstrate that DL methods can effectively generate synthetic data, utilize these data to train DL models, and simultaneously protect the privacy of the original real-world datasets.

## Results

### Patient and breast lesion characteristics

A total of 11,405 breast lesion US images in 6,011 patients (median age: 45) were collected from 202 hospitals that are members of the Chinese Artificial Intelligence Alliance for Thyroid and Breast Ultrasound across 29 provinces and municipalities of China. These included 5,050 images of BI-RADS 3, 2,649 images of BI-RADS 4A, 1,369 images of BI-RADS 4B, 1,662 images of BI-RADS 4C, and 675 images of BI-RADS 5. Table 1 depicts the patient and breast lesion characteristics for each dataset used in 3 tasks, namely, the deep generation task, the human evaluation task, and the classification task. The inclusion and exclusion of patients are documented in Fig. S1.

**Table 1.** Baseline patient and lesion characteristics. Except where indicated, data are number of patients or images, with percentages in parentheses.

Characteristics	Total group	Deep generation task	Human evaluation task	Classification task	
				Training set	Testing set
Overall images	11,405	9,705	500	800	400
Patients	6,011	5,243	279	331	158
Age (y)^a^	45 (36, 55)	45 (35, 55)	44 (35, 54)	44 (36, 55)	49 (38, 58)
BI-RADS 3 images	5,050 (44.28)	4,350 (44.82)	100 (20.00)	400 (50.00)	200 (50.00)
BI-RADS 4A images	2,649 (23.23)	2,399 (24.72)	100 (20.00)	100 (12.50)	50 (12.50)
BI-RADS 4B images	1,369 (12.00)	1,119 (11.53)	100 (20.00)	100 (12.50)	50 (12.50)
BI-RADS 4C images	1,662 (14.57)	1,412 (14.55)	100 (20.00)	100 (12.50)	50 (12.50)
BI-RADS 5 images	675 (5.92)	425 (4.38)	100 (20.00)	100 (12.50)	50 (12.50)

BI-RADS, Breast Imaging Reporting and Data System

^a^ Data are medians, with interquartile ranges in parentheses.

### Quality and privacy assessment of synthetic breast US images

**Fig. 2.**
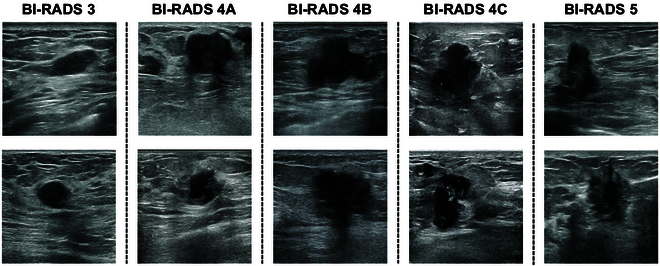
Examples of CoLDiT-generated breast ultrasound (US) images featuring different BI-RADS categories (including BI-RADS 3, 4A, 4B, 4C, and 5).

Our model systematically generated high-quality breast US images spanning diverse BI-RADS categories (BI-RADS 3, 4A, 4B, 4C, and 5) at a resolution of 512 × 512 pixels. Synthetic breast US images exemplifying BI-RADS 3, 4A, 4B, 4C, and 5 are depicted in Fig. 2. Qualitatively, the synthetic breast US images exhibit realistic textures and accurate anatomy, revealing multiple layers of tissue such as skin, fatty tissue, glandular tissue, and retromammary space, with lesions mostly situated in the glandular tissue. Moreover, when focusing on the quality of CoLDiT’s conditional generation, we observe that the synthetic images in Fig. 2 align well with their intended BI-RADS classification. This alignment is evident in various aspects, including the shape, orientation, margin, echo pattern, posterior features of lesions, and the architecture distortion of surrounding tissues such as Cooper’s ligaments.

To further assess the quality of synthetic breast US images, we acquired quantitative metrics, including IS and FID, for our model outputs. The IS metric quantifies both the quality and diversity of synthetic images, where higher scores demonstrate superior quality and diversity. The IS score for breast US images generated by CoLDiT is 2.32 (Table S1). The FID metric measures the distance between the distributions of real and generated images, with lower scores indicating greater similarity. Breast US images produced by CoLDiT demonstrate an FID score of 72.58 (Table S1).

To rule out the possibility that CoLDiT memorized and directly replicated images from the training set, we randomly sampled 500 synthetic breast US images and sought their nearest neighbors (Fig. S2) in the full training set using cosine similarity. A board-certified radiologist then evaluated the visual similarity between each synthetic image and its nearest neighbor to assess whether they appeared identical. Among the sampled synthetic breast US images, none were judged by the radiologist to be visually identical to their nearest neighbors in the training set. Therefore, we found no evidence of private information duplication from the training samples in the synthetic breast US images.

### Human evaluation of the realism of synthetic breast US images

**Fig. 3.**
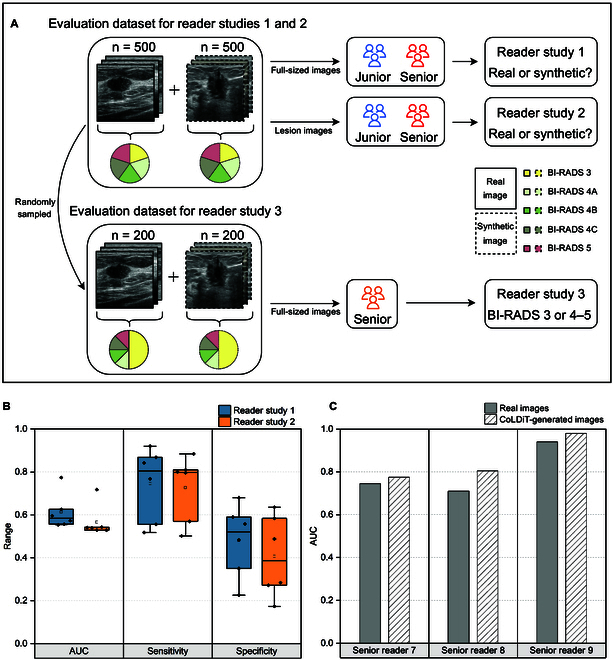
Procedures and results of human evaluation on CoLDiT-generated breast US images. (A) We evaluate CoLDiT-generated breast US images through 3 reader studies. Reader study 1 and reader study 2 assess the realism of CoLDiT-generated images, while reader study 3 evaluates the conditional generation of CoLDiT based on BI-RADS classification. (B) Evaluation performance of 6 readers regarding the realism of real and CoLDiT-generated breast US images in reader study 1 and reader study 2. (C) Comparison of each reader’s BI-RADS classification performance on real and CoLDiT-generated breast US images in reader study 3. AUC, area under the receiver operating characteristic curve.

To evaluate the realism of CoLDiT-generated breast US images, a panel of 6 readers, namely, the junior readers’ group (junior readers 1, 2, and 3) and the senior readers’ group (senior readers 4, 5, and 6), was presented with a set of 500 real and 500 synthetic breast US images in a randomized order. We conducted 2 independent reader studies, denoted as reader study 1 and reader study 2 (Fig. 3A). In reader study 1, 6 readers were instructed to assign labels of “real” or “synthetic” to each full-sized image (512 × 512 pixels) from the provided dataset. Two weeks later, in reader study 2, they were tasked with categorizing lesions cropped from images within the same dataset as either “real” or “synthetic”.

In reader studies 1 and 2, the area under the receiver operating characteristic curve (AUC) for 6 readers ranges from 0.53 to 0.77 (Table 2). Notably, junior reader 1 could not achieve a higher AUC than a random classifier in reader study 2 (AUC: 0.53 for junior reader 1 and 0.5 for the random classifier; *P* = 0.07). Although the remaining AUCs in reader studies 1 and 2 were significantly higher than 0.5 (*P* < 0.01), these AUCs were confined to a narrow range from 0.53 to 0.63, still close to 0.5, with the exception of senior reader 5 obtaining the highest AUCs of 0.77 and 0.72 in reader studies 1 and 2, respectively (Fig. 3B). The AUCs between the junior readers’ group and the senior readers’ group were comparable in both studies (*P* = 0.25 in reader study 1 and 0.75 in reader study 2). Upon comparing the AUCs of the same reader in reader studies 1 and 2, junior reader 2, senior reader 5, and senior reader 6 exhibited AUCs in reader study 1 that are superior to those in reader study 2 (*P* < 0.001 for 3 readers), while the remaining 3 readers demonstrated comparable AUCs in reader studies 1 and 2 (*P* = 0.16 for junior readers 1 and 3; *P* = 0.06 for senior reader 4).

**Table 2.** Performance of human readers in evaluating the realism of real and CoLDiT-generated breast US images. Data in parentheses for all metrics are 95% CIs. RS 1 designates reader study 1, which assesses the realism of full-sized breast US images. RS 2 designates reader study 2, which assesses the realism of lesion images cropped from full-sized ones.

Metrics for reader studies	Junior reader 1	Junior reader 2	Junior reader 3	Senior reader 4	Senior reader 5	Senior reader 6
**AUC**						
RS 1	0.56 (0.53, 0.59)	0.63 (0.60, 0.66)	0.55 (0.52, 0.58)	0.57 (0.54, 0.60)	0.77 (0.75, 0.80)	0.60 (0.57, 0.63)
RS 2	0.53 (0.50, 0.56)	0.54 (0.51, 0.57)	0.54 (0.51, 0.57)	0.54 (0.51, 0.57)	0.72 (0.69, 0.75)	0.53 (0.50, 0.56)
*P* value ^a^	0.16	<0.001	0.16	0.06	<0.001	<0.001
**Sensitivity (%)**						
RS 1	55.6 (51.1, 60.0)	76.8 (72.8, 80.4)	51.8 (47.3, 56.2)	92.0 (89.2, 94.2)	86.8 (83.4, 89.6)	84.2 (80.6, 87.2)
RS 2	57.0 (52.5, 61.4)	79.6 (75.7, 83.0)	50.2 (45.7, 54.7)	81.0 (77.2, 84.3)	80.0 (76.2, 83.4)	88.4 (85.2, 91.0)
**Specificity (%)**						
RS 1	55.8 (51.3, 60.2)	48.4 (43.9, 52.9)	59.0 (54.5, 63.3)	22.6 (19.0, 26.6)	68.0 (63.7, 72.0)	35.0 (30.8, 39.4)
RS 2	48.8 (44.3, 53.3)	28.4 (24.5, 32.6)	58.4 (53.9, 62.7)	27.2 (23.4, 31.4)	63.6 (59.2, 67.8)	17.4 (14.2, 21.1)

US, ultrasound; CI, confidence interval; AUC, area under the receiver operating characteristic curve

^a^ *P* values are for comparing the performance metrics between reader study 1 and reader study 2.

**Fig. 4.**
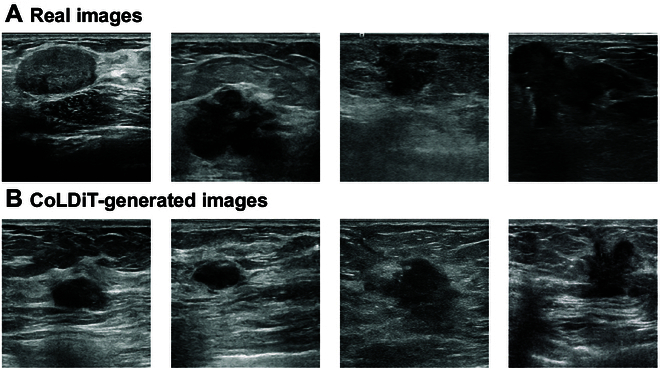
Examples of real and CoLDiT-generated breast US images labeled oppositely by at least 4 out of 6 readers in the realism evaluation. (A) Four examples of real breast US images that were labeled as synthetic by at least 4 out of 6 readers in the authenticity evaluation. (B) Four examples of CoLDiT-generated breast US images that were labeled as real by at least 4 out of 6 readers in the authenticity evaluation.

For all readers, the sensitivity, representing the capacity to distinguish real images from all real images, did not reach 100% in either reader study 1 (range: 51.8% to 92.0%) or reader study 2 (range: 50.2% to 88.4%) (Table 2 and Fig. 3B). Additionally, the specificity for the 6 readers ranges from 22.6% to 68.0% in reader study 1 and from 17.4% to 63.6% in reader study 2 (Table 2 and Fig. 3B). All 6 readers demonstrated lower specificity than sensitivity in both reader studies 1 and 2 (Table 2), suggesting that they more often misidentified synthetic images as “real”, rather than vice versa. Figure 4 displays examples of real and synthetic breast US images labeled oppositely by at least 4 out of 6 readers.

To test the internal consistency of readers’ assessments, we utilized the quantitative metric of Fleiss’ kappa. In reader study 1, there was slight consistency among all readers (Fleiss’ kappa = 0.132), as well as within the junior readers’ group (Fleiss’ kappa = 0.121) and the senior readers’ group (Fleiss’ kappa = 0.150), in differentiating between real and synthetic full-sized breast US images. However, we observed no consistency among readers in the evaluation of lesion images in reader study 2, as indicated by the negative values of Fleiss’ kappa for all readers (Fleiss’ kappa = −0.247), junior readers (Fleiss’ kappa = −0.559), and senior readers (Fleiss’ kappa = −0.867).

### Effectiveness of synthetic breast US images in recommending biopsy

According to the American College of Radiology BI-RADS, breast lesions classified as BI-RADS 4A or higher are recommended for biopsy [24]. Thus, to evaluate the effectiveness of synthetic breast US images in recommending biopsy, we engaged 3 additional radiologists, each possessing over 10 years of experience in breast US (referred to as senior readers 7, 8, and 9). In this reader study, designated as reader study 3, they were tasked with categorizing a mixture of 200 real and 200 synthetic breast US images into BI-RADS 3 or BI-RADS 4 to 5 (Fig. 3A). All images in the provided dataset are full sized, and the number of images in BI-RADS 3 versus BI-RADS 4 to 5 is equal, although unbeknownst to the readers.

The overall performance of the binary classification (BI-RADS 3 versus BI-RADS 4 to 5) on synthetic images is comparable to that on real images for all 3 senior readers, with senior reader 8 and senior reader 9 even surpassing the performance on real images. Specifically, senior reader 7 achieved an AUC of 0.745 for BI-RADS classification of real images and 0.775 for synthetic images (*P* = 0.42). For senior reader 8, the respective values were 0.710 and 0.805 (*P* = 0.006), while for senior reader 9, they were 0.940 and 0.980 (*P* = 0.04) (Table 3 and Fig. 3C).

**Table 3.** Performance of human readers in classifying the real and CoLDiT-generated breast US images into BI-RADS 3 or BI-RADS 4 to 5. Data in parentheses for all metrics are 95% CIs. All *P* values are for comparing the performance metrics between real and synthetic breast US images.

Readers and image types	AUC	Sensitivity (%)	Specificity (%)
**Senior reader 7**			
Real images	0.74 (0.68, 0.80)	97.0 (90.8, 99.2)	52.0 (41.8, 62.0)
Synthetic images	0.78 (0.71, 0.83)	98.0 (92.3, 99.6)	57.0 (46.7, 66.7)
*P* value	0.42	ND	ND
**Senior reader 8**			
Real images	0.71 (0.64, 0.77)	100 (95.4, 100)	42.0 (32.3, 52.3)
Synthetic images	0.80 (0.74, 0.86)	100 (95.4, 100)	61.0 (50.7, 70.4)
*P* value	0.006	ND	ND
**Senior reader 9**			
Real images	0.94 (0.90, 0.97)	95.0 (88.2, 98.1)	93.0 (85.6, 96.9)
Synthetic images	0.98 (0.95, 0.99)	98 (92.3, 99.6)	98 (92.3, 99.6)
*P* value	0.04	ND	ND

ND, no data

In the binary classification, BI-RADS 4 to 5 images were predefined as positive samples, and BI-RADS 3 images were predefined as negative samples. Hence, the sensitivity in Table 4 reflects readers’ classification accuracy on BI-RADS 4 to 5 images, while the specificity reflects their classification accuracy on BI-RADS 3 images. As shown in Table 4, senior readers 7, 8, and 9 accurately classified 97%, 100%, and 95% of real images as BI-RADS 4 to 5 and 98%, 100%, and 98% of synthetic images as BI-RADS 4 to 5, respectively. Moreover, senior readers 7, 8, and 9 accurately classified 52%, 42%, and 93% of real images as BI-RADS 3 and 57%, 61%, and 98% of synthetic images as BI-RADS 3, respectively.

**Table 4.** Comparison results of the performance of ResNet-50 trained with different training sets in classifying real breast US images into BI-RADS 3 or BI-RADS 4 to 5. Data in parentheses for all metrics are 95% CIs. Folds 1 to 5 originate from the 5-fold cross-validation using an external testing set.

Classifiers and folds	AUC	Sensitivity (%)	Specificity (%)	F1
**Classifier 1 ^a^**				
Average	0.95 ± 0.01	86.2 ± 1.1	96.0 ± 1.7	0.91 ± 0.003
Fold 1	0.94 (0.91, 0.96)	85.0 (79.1, 89.5)	97.0 (93.3, 98.8)	0.90 (0.78, 0.93)
Fold 2	0.95 (0.93, 0.97)	85.0 (79.1, 89.5)	98.5 (95.3, 99.6)	0.91 (0.88, 0.94)
Fold 3	0.96 (0.94, 0.98)	88.0 (82.5, 92.0)	93.5 (88.9, 96.3)	0.90 (0.88, 0.93)
Fold 4	0.94 (0.91, 0.96)	86.5 (80.8, 90.7)	95.0 (90.7, 97.4)	0.90 (0.87, 0.93)
Fold 5	0.94 (0.92, 0.96)	86.5 (80.8, 90.8)	96.0 (92.0, 98.1)	0.91 (0.88, 0.94)
**Classifier 2 ^b^**				
Average	0.95 ± 0.01	86.8 ± 1.6	94.8 ± 2.5	0.90 ± 0.004
Fold 1	0.96 (0.93, 0.97)	85.5 (79.7, 89.9)	97.0 (93.3, 98.8)	0.91 (0.88, 0.93)
Fold 2	0.95 (0.92, 0.97)	87.5 (81.9, 91.6)	92.5 (87.7, 95.6)	0.90 (0.87, 0.93)
Fold 3	0.94 (0.91, 0.96)	85.0 (79.1, 89.5)	97.0 (93.3, 98.8)	0.90 (0.87, 0.93)
Fold 4	0.95 (0.92, 0.97)	86.5 (80.8, 90.8)	96.5 (92.6, 98.4)	0.91 (0.88, 0.94)
Fold 5	0.96 (0.94, 0.98)	89.5 (84.2, 93.2)	91.0 (85.9, 94.4)	0.90 (0.87, 0.93)
***P* value ^c^**	0.81	0.58	0.46	0.81

^a^ Classifier 1 designates the ResNet-50 trained with 800 real breast US images, comprising 400 BI-RADS 3 and 400 BI-RADS 4 to 5 images.

^b^ Classifier 2 designates the ResNet-50 trained with 400 real and 400 CoLDiT-generated breast US images, encompassing 200 real BI-RADS 3, 200 real BI-RADS 4 to 5, 200 synthetic BI-RADS 3, and 200 synthetic BI-RADS 4 to 5 images.

^c^ *P* values are for comparing the performance metrics between classifier 1 and classifier 2.

### Performance of diagnostic classifiers trained on synthetic breast US images

**Fig. 5.**
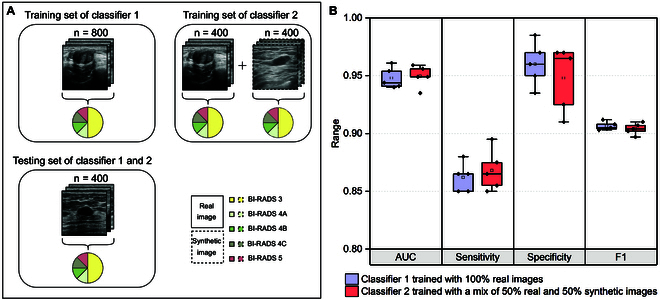
CoLDiT-generated breast US images effectively augment the training data of the BI-RADS classification task, achieving performance comparable to that using a training set comprising solely of real images. (A) Displayed are the training sets and the testing set utilized for 2 classifiers. The testing set remains consistent across both classifiers, while the training sets differ. Classifier 1 utilizes 100% real images, whereas classifier 2 employs a mix of 50% real and 50% synthetic images. (B) A comparison of the performance of 2 classifiers in classifying real breast US images into BI-RADS 3 or BI-RADS 4 to 5.

To explore the potential clinical applicability of synthetic data, we applied it to the task of classifying breast US images into BI-RADS 3 or BI-RADS 4 to 5 using the ResNet-50 architecture. We utilized 2 distinct datasets for model training: one comprising 800 real breast US images and the other consisting of 400 real and 400 CoLDiT-generated breast US images. We conducted 5-fold cross-validation to assess the stability of the model’s performance across different data subsets (Table S2). Subsequently, we employed an external testing set of 400 real breast US images to test each model’s performance on real-world data. We calculated AUC, sensitivity, specificity, and F1 scores to evaluate model performance. The breast lesion characteristics of training and testing sets are illustrated in Fig. 5A and Table 1.

The classifier trained with a mix of 50% real and 50% synthetic breast US images exhibited performance comparable to that trained solely with real breast US images, with *P* values being 0.81 for AUC, 0.58 for sensitivity, 0.46 for specificity, and 0.81 for F1 scores (Fig. 5B). Specifically, for the classifier trained solely with real breast US images (referred to as classifier 1 in Table 4), the average values of AUC, sensitivity, specificity, and F1 scores across 5 folds were 0.948, 86.2%, 96.0%, and 0.906, respectively. For the classifier trained with a mix of 50% real and 50% synthetic breast US images (referred to as classifier 2 in Table 4), the corresponding values were 0.950, 86.8%, 94.8%, and 0.904, respectively. Detailed performance metrics for each fold are presented in Table 4. Overall, replacing half of the real data with synthetic data yielded a performance in the BI-RADS classification task similar to that using an equal-sized training set comprising solely real images.

## Discussion

Medical big data are revolutionizing healthcare. However, medical data sharing across multiple centers, essential for building large and diverse datasets, raises privacy concerns and the risk of personal information misuse. One approach to enabling medical data sharing while preserving privacy is to generate synthetic datasets that closely resemble the original data. Our work focuses on the synthesis of medical imaging data. We introduce CoLDiT, a deep generative model for medical image synthesis, with breast US image synthesis as our initial exploration. Our results demonstrate that CoLDiT can generate realistic breast US images across various BI-RADS categories, offering a promising solution for medical data sharing without compromising privacy.

CoLDiT is a conditional LDM with a DiT backbone for high-resolution breast US image generation. The state-of-the-art performance of DMs in natural image synthesis [16] has sparked interest in their application to US image generation. Previous works have primarily focused on generating echocardiography images and videos conditioned on semantic label maps, texts, or interpretable clinical parameters such as left ventricle ejection fractions [25–27]. Other studies explored generating musculoskeletal, lung, and liver US images using DM-based methods [28–30]. To the best of our knowledge, only one study has applied DMs to breast US image synthesis, utilizing US low-rank adaptation to fine-tune the text-guided Stable Diffusion model [31].

Our study offers several advantages over prior works. First, we used a large multicenter dataset of 9,705 breast US images for CoLDiT training, ensuring diverse data sources across different vendors or grades from 202 hospitals. This allows the model to capture a comprehensive range of variations inherent in real-world breast US images, leading to the generation of more realistic and precise synthetic images. Second, we employed a pure transformer backbone in our LDM, instead of the traditional U-Net backbone. Transformers, renowned for their exceptional ability to capture long-range dependencies, enable our models to generate more coherent and detailed images. Third, we conditioned breast US image synthesis on BI-RADS labels, allowing the generation of US images corresponding to specific BI-RADS categories. This is particularly valuable in medical contexts, where the ability to generate images tailored to specific clinical scenarios is crucial for accurate diagnosis and treatment planning.

A comprehensive evaluation of synthetic data should account for their ability to avoid replicating private information, maintain realism, and ensure utility in practical applications. We conducted assessments on CoLDiT-generated breast US images based on the aforementioned 3 criteria.

Privacy leakage is a major concern in medical data sharing, including when deep generative models are used for this purpose. Carlini et al. [32] showed that DMs can memorize individual training images and reproduce them during generation. However, in our privacy assessment of synthetic breast US images, we found no duplication of training samples in the randomly selected subset of synthetic images. We hypothesize that this may be attributed to the unique characteristics of US images, which, unlike natural images, exhibit lower contrast, higher noise levels, and more complex and ambiguous structural features. These properties likely push the model to learn the overall data distribution and high-level features rather than memorizing specific samples. Furthermore, the DiT backbone’s ability to capture global context might have contributed to the reduced risk of overfitting to specific training samples, further minimizing the likelihood of memorization. Nevertheless, this remains a hypothesis, and further studies are required to explore this effect across different datasets and model architectures. Furthermore, we employed the CLIP (Contrastive Language-Image Pretraining) ViT-B/32 encoder, which is pretrained on natural images to extract feature vectors from US images. Although CLIP’s extensive training datasets offer potential generalization capabilities for capturing the structural and textural features of US images, further research is necessary to validate its effectiveness across different types of medical images. Additionally, developing encoders tailored to medical imaging using large-scale, domain-specific datasets could provide more accurate and reliable feature representations.

In the quantitative evaluation of CoLDiT-generated images, we obtained a score of 2.32 for IS and 72.58 for FID, deemed acceptable for breast US images synthesis in this study. It is worth noting that both metrics are founded on Inception V3, which is pretrained on the natural image dataset ImageNet. Therefore, the efficacy of these 2 metrics in assessing the quality of synthetic US images remains uncertain, and the benchmark ranges for natural image generation can hardly be referenced. Here, we reported these 2 metrics to serve as references for subsequent research on breast US image generation, despite flaws in these metrics. For further assessment, we conducted reader studies and downstream tasks to demonstrate that CoLDiT effectively generated high-quality breast US images conditioned on BI-RADS categories.

The realism of synthetic breast US images was assessed through 2 reader studies: one evaluating full-sized images (reader study 1) and the other examining cropped lesion images (reader study 2). For 5 readers (excluding senior reader 5), the AUCs in both studies ranged from 0.53 to 0.63, highlighting the difficulty in distinguishing real from synthetic images. In contrast, senior reader 5 achieved higher AUCs of 0.77 and 0.72 in reader studies 1 and 2, respectively. This discrepancy may be attributed to differences in radiologists’ expertise and their varying prior knowledge of how synthetic images are expected to appear. When comparing the same reader’s performance across both studies, 3 out of 6 readers (junior reader 2, senior reader 5, and senior reader 6) attained higher AUCs in reader study 1 than in reader study 2 (*P* < 0.001). This suggests that the tissue structures surrounding breast lesions may influence readers’ judgments, providing additional, possibly unrealistic evidence for synthetic image assessment.

All 6 readers failed to recognize all real images in reader study 1 (sensitivity: 51.8% to 92.0%) and reader study 2 (sensitivity: 50.2% to 88.4%), implying that structures confusingly resembling synthetic ones can also appear in real breast US images. This may be due to lesions with uncommon shapes, structures, or anatomical positions, which do not align with typical cognitive patterns of many radiologists. Specificity was lower than sensitivity for all readers in both studies. This suggests that synthetic images generally feature accurate structures with minimal artifacts, resulting in a lack of evidence for labeling images as synthetic, hence yielding low specificity. Slight and no consistency among readers (Fleiss’ kappa <0.1 for reader study 1 and <0 for reader study 2) further demonstrate the challenge for readers in distinguishing between real and synthetic images, emphasizing the realism of CoLDiT-generated images.

To further validate the utility of synthetic breast US images for biopsy recommendations, another reader study was conducted, with 3 senior readers classifying each image from a mixture of real and synthetic images as BI-RADS 3 or 4 to 5. The AUC was comparable between real images and synthetic images (*P* = 0.42 for senior reader 7), with senior reader 8 and senior reader 9 even obtaining a higher AUC on synthetic images (*P* = 0.006 and 0.04, respectively). The gold standard for the BI-RADS classification of real images was based on assessments by radiologists with over 20 years’ experience in breast US, while for synthetic images, it was based on CoLDiT. The similar AUCs between real and CoLDiT-generated images suggest that CoLDiT’s control over conditional generation of BI-RADS 3 and BI-RADS 4 to 5 images is comparable to the assessment of experienced radiologists.

We explored CoLDiT’s effectiveness in augmenting BI-RADS classification training sets by replacing half of the real data with CoLDiT-generated data for model training. This approach yielded AUCs, sensitivity, specificity, and F1 scores comparable to those using solely real data (*P* > 0.05 for all). This suggests that synthetic images may introduce more meaningful variations that help generalize classifiers to unseen data, thereby mitigating the gap between synthetic and real data. However, synthetic images may also introduce unrealistic features learned by classifiers, necessitating further research to ensure the reliable use of synthetic data.

This study has several limitations. First, medical big data have diverse sources, each playing a crucial role at different stages of medical decision-making. We employed only B-mode breast US images to develop the generative model in this study. Future work could explore the broader utility of this approach in synthesizing other forms of medical data. Second, the BI-RADS classifications of all real breast US images relied on the subjective assessments made by the original interpreting radiologists during clinical practice. Although the final BI-RADS category of each real image was checked by radiologists with over 20 years’ experience in breast US, individual biases about BI-RADS categories were still inevitable.

In summary, we present CoLDiT, a deep generative model for synthesizing high-quality breast US images conditioned on BI-RADS categories. CoLDiT-generated images can augment the training dataset of DL models, yielding performance comparable to that obtained by an equal-sized training set composed exclusively of real images. Our results demonstrate that synthetic data hold promise as a privacy-preserving solution, overcoming barriers to medical data sharing and advancing the secure utilization of medical big data.

## Materials and Methods

### Datasets and preprocessing

We conducted a retrospective study at 202 hospitals that are members of the Chinese Artificial Intelligence Alliance for Thyroid and Breast Ultrasound across 29 provinces and municipalities in China. The study was approved by the institutional review board of the primary study center (Ruijin Hospital). Written informed consent was acquired from patients before US examinations. Patients with breast lesions who underwent diagnostic breast US examination from November 2017 to January 2024 and had corresponding surgical pathological evaluation of their breast lesions were included. The detailed inclusion and exclusion criteria can be found in Appendix S1.

The breast US examinations were performed by board-certified radiologists with at least 5 years of experience in the field, using US systems of 11 different vendors with varying grades (Appendix S1). These US systems were equipped with high-frequency linear probes. At least one longitudinal US image and one transverse image of the target lesion were stored. All US images, BI-RADS categories, relevant clinical information, and pathological results were collected and forwarded to Ruijin Hospital for further processing. Although the data included grayscale images, Doppler images, and cine clips, this study focused exclusively on grayscale images. In total, we adopted 11,405 grayscale breast lesion US images from 6,011 different patients, with patient and breast characteristics depicted in Table 1. The inclusion and exclusion of patients are documented in Fig. S1. Subsequently, a total of 11,405 grayscale images were divided into 3 distinct datasets for different experiments using stratified sampling to ensure representative distribution across various patient characteristics. The first dataset used for training the deep generation model of CoLDiT included 9,705 images. The second dataset of 500 images formed the real image dataset of human evaluation tasks. The third dataset used for the classification task included 1,200 images, with 800 images for training and 400 images for external testing, using 5-fold cross-validation. The patient and breast characteristics of 3 datasets are depicted in Table 1. Before using each dataset, we resized each US image to a size of 512 × 512 pixels and applied a mean normalization to all images within each dataset.

### Methods for developing CoLDiT

#### Utilizing BI-RADS categories as conditions for CoLDiT

The BI-RADS lexicon, established by the American College of Radiology, standardizes breast US templates and provides clinical management recommendations (Table S3). We generated breast US images conditioned on BI-RADS categories for 2 primary reasons. First, BI-RADS offers an additional criterion for evaluating synthetic breast US images beyond image realism, resulting in greater assessability and reliability than generating images without constraints. Second, conditional generation based on BI-RADS classification broadens the potential utility of synthetic breast US images. Given the variability in BI-RADS ratings among observers due to differences in expertise and interpretations, synthetic breast US images could serve as valuable training data for BI-RADS classification models and educational resources for medical students and resident radiologists. Therefore, incorporating BI-RADS classification into the conditional generation of breast US images offers substantial clinical and educational benefits.

#### Development of CoLDiT

Our model CoLDiT is based on DiT [23] as shown in Fig. 1. Following DiT, we used the LDM framework [18], where the training process involves 2 stages: (a) training an autoencoder to encode images to latent representatives with a learned encoder and (b) training a DM on the latent representatives. In the first training phase, we used the pretrained VAE model from Stable Diffusion [18] where an image of 512 × 512 × 3 resolution could be compressed into a latent representative $z_{0}$ of 64 × 64 × 4 resolution with a downsampling factor of 8. In the second training phase, we trained a DM using DiT-S as the backbone, where “S” denotes the small model size used by DiT. Specifically, we first applied noise to $\begin{array}{c} z_{0}:\left(z_{t}|z_{0}\right)=\text{N}\left(z_{t};\sqrt{\left({\bar{\alpha }}_{t}\right)}z_{0},\left(1-{\bar{\alpha }}_{t}\right)\text{I}\right) \end{array}$, where I is the identity matrix, with the reparameterization trick to obtain $z_{t}$ as follows: $z_{t}=\sqrt{{\bar{\alpha }}_{t}}z_{0}+\sqrt{1-{\bar{\alpha }}_{t}}\epsilon_{t}$, where ${\bar{\alpha }}_{t}$ are hyperparameters and *ϵ_t_* ~ Ν(0, I). Then, the model was trained to learn the reverse diffusion process, where neural networks were used to predict the added noise of each timestep *t*, *t* = 1, …, *T*. Note that, instead of using U-Net as the backbone of the noise predictor, we used the DiT architecture. During the reverse diffusion process, we first applied patchify to extract a sequence of patch embeddings from the noisy latent representation of $z_{t}$, *t* = 1, …, *T*, with a patch size of 2. The input tokens of the DiT block involved patch embeddings of the noisy latent, as well as embeddings of the timestep and the label of BI-RADS classification. Each DiT block followed the structure of the adaLN-Zero block [23]. After the final DiT block, we operated the final adaptive layer norm and then linearly transform each token into a tensor with a size of 2 × 2 × 8. In the final step, we rearranged the decoded tokens into their original spatial layout to obtain the predicted noise and covariance results. The DM was trained with the simple mean squared error (MSE) between the predicted noise $\epsilon_{\theta }\left(z_{t}\right)$ and the ground truth sampled Gaussian noise $\epsilon_{t}$: $L_{\text{simple}}\left(\theta \right)={\left\|\epsilon_{\theta }\left(z_{t}\right)-\epsilon_{t}\right\|}_{2}^{2}$.

Once the DM was trained, new images of different BI-RADS categories could be generated by sampling representations from the DM using the denoising diffusion implicit model (DDIM) sampler [17] and then decoding them to images with the learned VAE decoder. During the sampling process, we employed classifier-free guidance to enhance the quality of generated images [19]. Classifier-free guidance in generative models involves conditioning the generation process without relying on explicit classifiers, where a conditional and an unconditional DM are jointly trained. The sampling can be established as follows: $\begin{array}{c} \tilde{\epsilon_{\theta }}\left(z_{t},c\right)=\left(1+\omega \right)\epsilon_{\theta }\left(z_{t},c\right)-\omega \epsilon_{\theta }\left(z_{t}\right) \end{array}$, where ω is a parameter that controls the strength of the implicit classifier and *c* is the conditioning information. The modified score $\tilde{\epsilon_{\theta }}\left(z_{t},c\right)$ is then used in place of $\epsilon_{\theta }\left(z_{t},c\right)$ when sampling from the DM.

In this study, we adopted latent space manipulations to guide the conditional generation: $z_{c}=z⊕c$, in which $z_{c}$ represents the conditioned latent vector, *z* represents the original latent vector sampled from an unconditional diffusion, *c* represents the conditioning information (e.g., the BI-RADS category labels), and ⊕ denotes the modulation operation used to combine *z* and *c*. Then, we applied conditioning operations on latent space and decoding networks: $z_{c}=f_{\text{encode}}\left(x_{\text{cond}}\right)$ and $x=f_{\text{decode}}\left(z_{c}\right)$, where *x* is the conditional output, *x*_cond_ is the conditional input to the decoding network, *f*_encode_ is the encoding function, and *f*_decode_ is the decoding function.

#### Training and sampling details of CoLDiT

We trained CoLDiT using a dataset comprising 9,705 breast US images. At the preprocessing stage, we resized each US image to 512 × 512 by default. For DiT settings, we initialized the final linear layer with zeros, while standard weight initialization techniques from ViT were applied elsewhere [21]. We employed the AdamW optimizer with a fixed learning rate of 1 × 10^−4^ and no weight decay. The batch size was set to 256. We applied horizontal flips for training data augmentation. Following previous methods [23], we utilized an exponential moving average of DiT weights during training, with a decay rate of 0.9999. We kept diffusion hyperparameters from Dhariwal and Nichol [16]. We used a DDIM sampler with 250 inference steps and the classifier-free guidance with a guidance scale of 0.8. The computational cost of CoLDiT amounts to approximately 10,000 graphics processing unit (GPU)-hours on an NVIDIA GPU.

### Methods for evaluating synthetic breast US images

#### Image quality

We used established metrics, including IS and FID, to quantitatively evaluate the quality of images generated by our model CoLDiT. The IS metric is computed using the logit outputs of an Inception v3 network pretrained on ImageNet-1k [33]. The higher IS scores of synthetic images demonstrate higher quality via a lower entropy of the label distribution and better diversity via a more uniform distribution of labels. Specifically, the IS score is defined as follows: $\text{IS}\left(G\right)=\exp\left(E_{x∼p_{g}}D_{\mathrm{KL}}\left(p\left(y|x\right)\|p\left(y\right)\right)\right)$, where $x∼p_{g}$ is an image sampled from $p_{g}$, $D_{\mathrm{KL}}\left(p\|q\right)$ is the Kullback–Leibler divergence between the distributions *p* and *q*, $p\left(y|x\right)$ is the conditional class distribution, and $p\left(y\right)=\int_{x}p\left(y|x\right)p_{g}\left(x\right)$ is the marginal class distribution. The exp in the expression is included to facilitate easier comparison of the values, so it will be ignored and we will use $\ln\left(\text{IS}\left(G\right)\right)$ without loss of generality.

The FID metric measures the similarity between the distribution of real images and generated images in the feature space, with a lower FID indicating higher similarity [34]. Specifically, we obtained the feature representations for both real and generated images after the last pooling layer of the Inception v3 network and then calculated the FID score according to $\begin{array}{c} \begin{array}{c} d^{2}\left(F,G\right)={\left|\mu_{X}-\mu_{Y}\right|}^{2}+\mathrm{tr}\left[\sum_{X}+\sum_{Y}-2{\left(\sum_{X}\sum_{Y}\right)}^{1/2}\right] \end{array} \end{array}$, where $\mu_{X},\mu_{Y}$ and $\sum_{X},\sum_{Y}$ are the respective means and covariance matrices of *F* and *G*, and the positive square root is taken. The above formula holds in particular when *F* and *G* are normal distributions on $R^{n}$. Additionally, it will be seen that $d_{0}^{2}\left(\sum_{X},\sum_{Y}\right)=\mathrm{tr}\left[\sum_{X}+\sum_{Y}-2{\left(\sum_{X}\sum_{Y}\right)}^{1/2}\right]$ defines a metric on the space of all covariance matrices of order *n*.

#### Image privacy

To assess the similarity between synthetic breast US images and the training samples, we identified the nearest neighbors in the training set for a randomly selected subset of 500 synthetic images. Using the CLIP ViT-B/32 encoder, we extracted 512-dimensional feature vectors for each image and computed the cosine similarity between each synthetic image and all training images [35,36]. The nearest neighbor was determined based on the minimal cosine distance. A board-certified radiologist then evaluated the visual similarity between each synthetic image and its nearest neighbor to assess whether they appeared identical.

#### Reader study

To evaluate the authenticity of synthetic breast US images, we engaged 6 readers, comprising 3 radiology residents (referred to as the junior readers’ group: junior readers 1, 2, and 3) and 3 board-certified radiologists with over 10 years of experience in breast US (referred to as the senior readers’ group: senior readers 4, 5, and 6). A breast US dataset of 500 real and 500 synthetic images was sampled from the real dataset and the generated dataset, respectively. The proportions of BI-RADS 3, 4A, 4B, 4C, and 5 for both real and synthetic images are equally distributed. These images were presented to the readers in a random order, with the readers uninformed about the dataset’s composition and instructed to evaluate all images on the same monitor. We conducted 2 independent reader studies using the same US imaging dataset prepared earlier. Both reader studies aim at assessing images’ authenticity, albeit differing as follows: the initial reader study (referred to as reader study 1) tasked readers with evaluating the authenticity of each full-sized image (512 × 512 pixels) in the provided dataset, classifying each image as real or synthetic, whereas the subsequent study (referred to as reader study 2) focused on authenticating lesion images cropped from full-sized images, with all other procedures being identical. The intervals between 2 studies were 2 weeks, and the sequence of images was rerandomized for each study. For each labeled set, we calculated the metrics of AUC, sensitivity, and specificity to evaluate the performance of each reader. Furthermore, Fleiss’ kappa was determined to assess interparticipant agreement. We used the following level definitions for Fleiss’ kappa values: 0.81 to 1.00 indicates almost perfect agreement, 0.61 to 0.80 indicates substantial agreement, 0.41 to 0.60 indicates moderate agreement, 0.21 to 0.40 indicates fair agreement, 0.01 to 0.20 indicates slight agreement, and 0 or less indicates no agreement.

To evaluate the effectiveness of CoLDiT-generated breast US images in recommending biopsy, we enlisted 3 additional radiologists with over 10 years of experience in breast US (referred to as senior readers 7, 8, and 9). This reader study (referred to as reader study 3) tasked 3 readers with categorizing 200 real and 200 synthetic breast US images into BI-RADS 3 and BI-RADS 4 to 5. All images in the provided dataset were full sized, and the number of images in BI-RADS 3 versus BI-RADS 4 to 5 is equal, although unbeknownst to the readers. For each labeled set, we computed the metrics of AUC, sensitivity, and specificity to evaluate the performance of each reader.

### Methods for developing BI-RADS classification models

We utilized 2 distinct datasets for model training: training set 1 comprising 800 real breast US images and training set 2 consisting of 400 CoLDiT-generated breast US images and 400 real images that were randomly sampled from training set 1. We adopted ResNet-50 [37], which uses a bottleneck design for its building blocks, to predict the BI-RADS category (BI-RADS 3 or BI-RADS 4 to 5) of breast US images. During the training process, ResNet-50 parameters were iteratively updated using backpropagation, and the cross-entropy loss function was employed. We conducted 50 epochs of training with a batch size of 32, and the learning rate was maintained at a constant of 1 × 10^−4^, without the application of weight decay. The AdamW stochastic method was used as the optimization method. We conducted 5-fold cross-validation to assess the stability of each model’s performance across different data subsets. Four folds were used for training, while one fold was reserved for internal testing, with iterations ensuring the coverage of the entire database for training and internal testing conditions. We then employed a fixed external testing set of 400 real breast US images to test each model’s performance on real-world data. We calculated the metrics of AUC, sensitivity, specificity, and F1 scores to evaluate model performance. The characteristics of breast lesion in training and testing sets are depicted in Fig. 5A and Table 1. The computational cost of ResNet-50 amounts to 40 GPU-hours on an NVIDIA GPU.

### Statistical analysis

Descriptive statistics were summarized as either mean ± SD or median. The Wilcoxon signed-rank test was utilized to compare the AUCs of junior and senior readers in reader study 1 and reader study 2, as well as the AUCs of 2 classifiers with 5-fold cross-validation. Otherwise, the Delong test was applied for AUC comparisons. Additionally, the Wilcoxon signed-rank test was also utilized to compare the sensitivity, specificity, and F1 scores of 2 classifiers with 5-fold cross-validation. *P* < 0.05 was considered to have a significant difference. Statistical analyses were conducted using MedCalc (version: 20.010) and Python (version: 3.7.6).

## Data Availability

All data from this study are available through the Department of Ultrasound at Ruijin Hospital. Requests for academic use of raw data can be addressed to the corresponding authors. All requests will be reviewed promptly to assess any intellectual property or patient confidentiality concerns and will be processed in accordance with institutional and departmental guidelines, requiring a material transfer agreement. All source code will be available upon request.
