# Supplementary material for: Synthetic Breast Ultrasound Images: A Study to Overcome Medical Data Sharing Barriers
Source: Research (Wash D C). 2024 Dec 3;7:0532. doi: 10.34133/research.0532 (PMC11612121; doi:10.34133/research.0532)
Supplement: Supplementary 1 — Appendix S1 Figs. S1 and S2 Tables S1 to S3 [file research.0532.f1.zip › figS1.pdf]

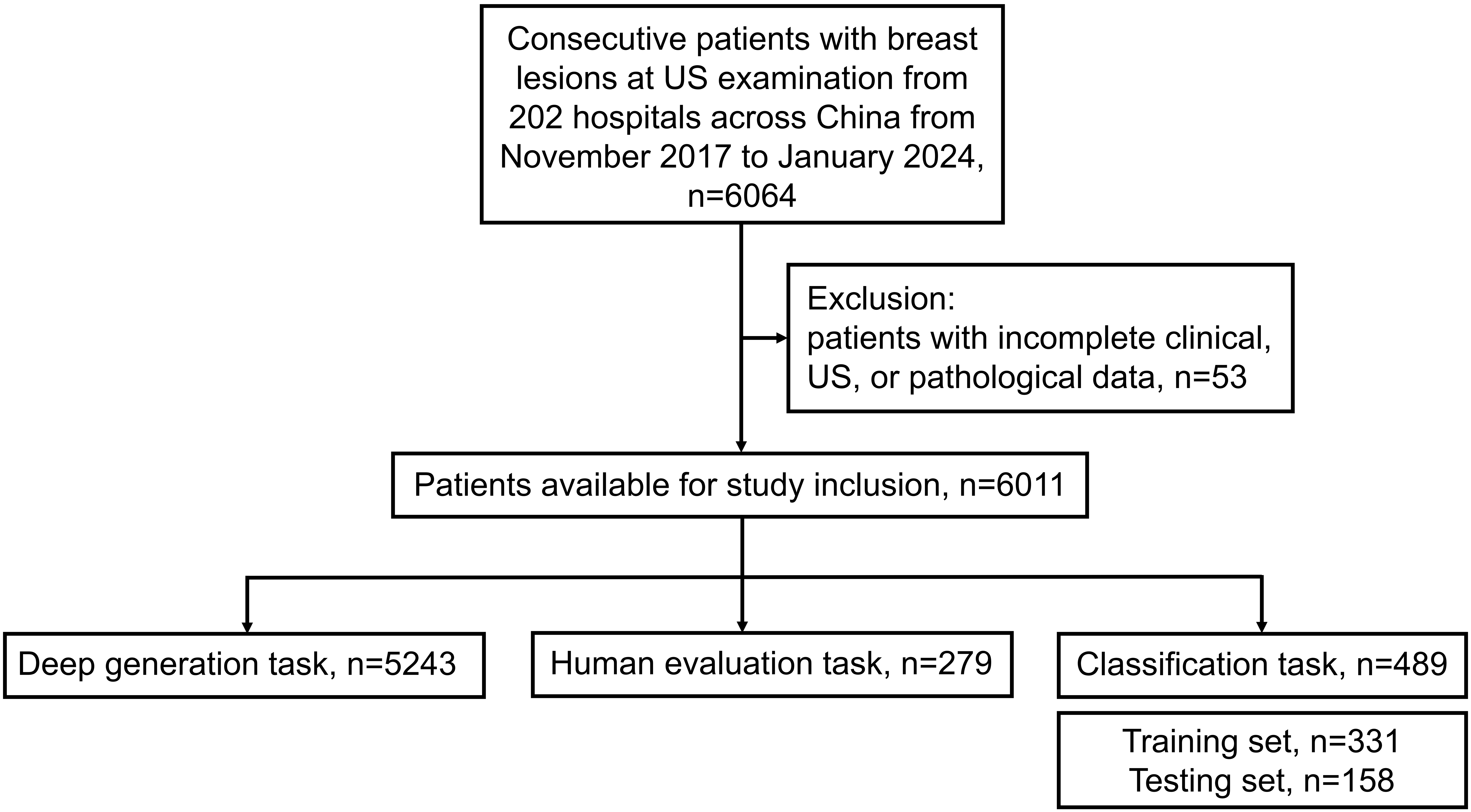
Consecutive patients with breast lesions at US examination from 202 hospitals across China from November 2017 to January 2024, n=6064
Exclusion:
patients with incomplete clinical, US, or pathological data, n=53
Patients available for study inclusion, n=6011
Deep generation task, n=5243
Human evaluation task, n=279
Classification task, n=489
Training set, n=331
Testing set, n=158
