# Supplementary material for: Synthetic Breast Ultrasound Images: A Study to Overcome Medical Data Sharing Barriers
Source: Research (Wash D C). 2024 Dec 3;7:0532. doi: 10.34133/research.0532 (PMC11612121; doi:10.34133/research.0532)
Supplement: Supplementary 1 — Appendix S1 Figs. S1 and S2 Tables S1 to S3 [file research.0532.f1.zip › Supplementary Materials.docx]

# Supplementary Materials

**Appendix S1**

**Patient Data Collection**

Our study included 202 hospitals from CAAU, including 148 tertiary level A hospitals, 25 tertiary level B hospitals, 27 secondary level A hospitals, and 2 secondary level B hospitals. In China, tertiary level A hospitals represent those with the highest level of medical care, followed by tertiary level B hospitals, secondary level A hospitals, and secondary level B hospitals.

A series of commercially available US systems were used in our study as follows: Esaote Class C, DU6, MyLab 60, MyLab 65, MyLab 75, MyLab 90, MyLab Twice (Esaote, Genoa, Italy); GE LOGIQ E8, LOGIQ E9, LOGIQ 9, LOGIQ E10, LOGIQ E11, LOGIQ E20, LOGIQ E22, LOGIQ P6, LOGIQ P9, LOGIQ S8, Vivid E9, Vivid E10, Voluson E6, Voluson E8, Voluson E9, Voluson S6, Voluson S8 (GE Medical Systems, Milwaukee, WI, USA); Hitachi EUB-8500, VISION Avius, VISION Ascendus, VISION Preirus, Arietta 70; Aloka Pro Sound SSD-3500, Alpha 7, Alpha 10 (Hitachi-Aloka Medical, Tokyo, Japan); Sumsung RS80A, (Samsung Medison, Seoul, Korea); Mindray DC-7, DC-8s, DC-80, DC-8exp, M9, Resona 7, Resona 7T, Resona 7S, Resona 8, Resona 9 (Mindray Medical International, Shenzhen, China); Philips Affiniti 50, Affiniti 70, Envisor, EPIQ 5, EPIQ 5C, EPIQ 7, EPIQ 7C, iE33, iE ELITE, iU22, iU ELITE, HD11 XE, HD15 (Philips Medical Systems, Best, the Netherlands); Siemens ACUSON NX3, ACUSON Oxana2, ACUSON Oxana3, ACUSON X700, S2000, S3000, Sequia 512 (Siemens Medical Solutions, Mountain View, CA, USA); SonoScape S50, S8 Exp (SonoScape Medical Corp, Shenzhen, China). SuperSonic Aixplorer (SuperSonic Imagine, Aix-en-Provence, France); and Toshiba Aplio 300, Aplio 400, Aplio 500 (Toshiba Medical Systems Corp., Tokyo, Japan);

All participating radiologists were trained in standardized breast ultrasound US examination methods. When performing US examinations, the radiologists were not blinded to patient history, complaints, and risk factors. All imaging data was stored on the local hard drive or PACS system.

**Inclusion and Exclusion Criteria**

The inclusion criteria were: (1) Age > 18 years old; (2) Preoperative US examination should display breast lesions comprehensively and obtain grayscale US images, Doppler images, and cine clips, with satisfactory image quality; (3) The surgical treatment or core needle biopsy should be performed within one week after the US examination to obtain the pathology diagnosis; (4) For patients with benign breast lesions or BI-RADS 3 lesions, long-term follow-up of at least three years is required; (5) Pathological results should correspond to the breast lesions identified by US examinations.

The exclusion criteria were: (1) Patients who have breast surgery history or breast implants; (2) Patients who have undergone biopsy in another institution prior to US evaluation of breast lesions; (3) Patients with breast cancer who have received neoadjuvant chemotherapy or preoperative treatment; (4) Patients who are pregnant or lactating; (5) Patients with incomplete clinical, US, or pathological data.

**Table S1: Quantitative metrics for evaluating breast US images generated by CoLDiT.**

|  | **Overall** | **BI-RADS category of synthetic images** | | | | |
| --- | --- | --- | --- | --- | --- | --- |
|  |  | **3** | **4A** | **4B** | **4C** | **5** |
| IS ↑ | 2.32±0.13 | 2.11±0.12 | 2.29±0.25 | 1.92±0.18 | 1.96±0.20 | 1.94±0.13 |
| FID ↓ | 72.58 | 85.25 | 126.46 | 117.48 | 132.98 | 228.98 |

**Table S2: Internal validation results of two classifiers in five-fold cross-validation.**

| AUC of | Fold | | | | | Mean | Variance |
| --- | --- | --- | --- | --- | --- | --- | --- |
|  | 1 | 2 | 3 | 4 | 5 |  |  |
| Classifier 1^a^ | 0·935 | 0·960 | 0·913 | 0·903 | 0·961 | 0·934 | 0·0007 |
| Classifier 2^b^ | 0·989 | 0·975 | 0·985 | 0·956 | 0·958 | 0·973 | 0·0002 |

Note.— AUC = area under the receiver operating characteristic curve

^a^Classifier 1 designates the ResNet-50 trained with 800 real breast US images, comprising 400 BI-RADS 3 and 400 BI-RADS 4-5 images.

^b^Classifier 2 designates the ResNet-50 trained with 400 real and 400 CoLDiT-generated breast US images, encompassing 200 real BI-RADS 3, 200 real BI-RADS 4-5, 200 synthetic BI-RADS 3, and 200 synthetic BI-RADS 4-5 images.

**Table S3: BI-RADS assessment categories for breast US images.**

| BI-RADS Category | Assessment |
| --- | --- |
| Category 0 | Incomplete — Need additional imaging evaluation |
| Category 1 | Negative |
| Category 2 | Benign |
| Category 3 | Probably Benign (> 0% but ≤ 2% likelihood of malignancy) |
| Category 4 | Suspicious (> 2% but < 95% likelihood of malignancy) |
|  | Category 4A: Low suspicion for malignancy (> 2% but ≤ 10% likelihood of malignancy) |
|  | Category 4B: Moderate suspicion for malignancy (> 10% but ≤ 50% likelihood of malignancy) |
|  | Category 4C: High suspicion for malignancy (> 50% but < 95% likelihood of malignancy) |
| Category 5 | Highly suggestive of malignancy (≥ 95% likelihood of malignancy) |
| Category 6 | Known biopsy-proven malignancy |


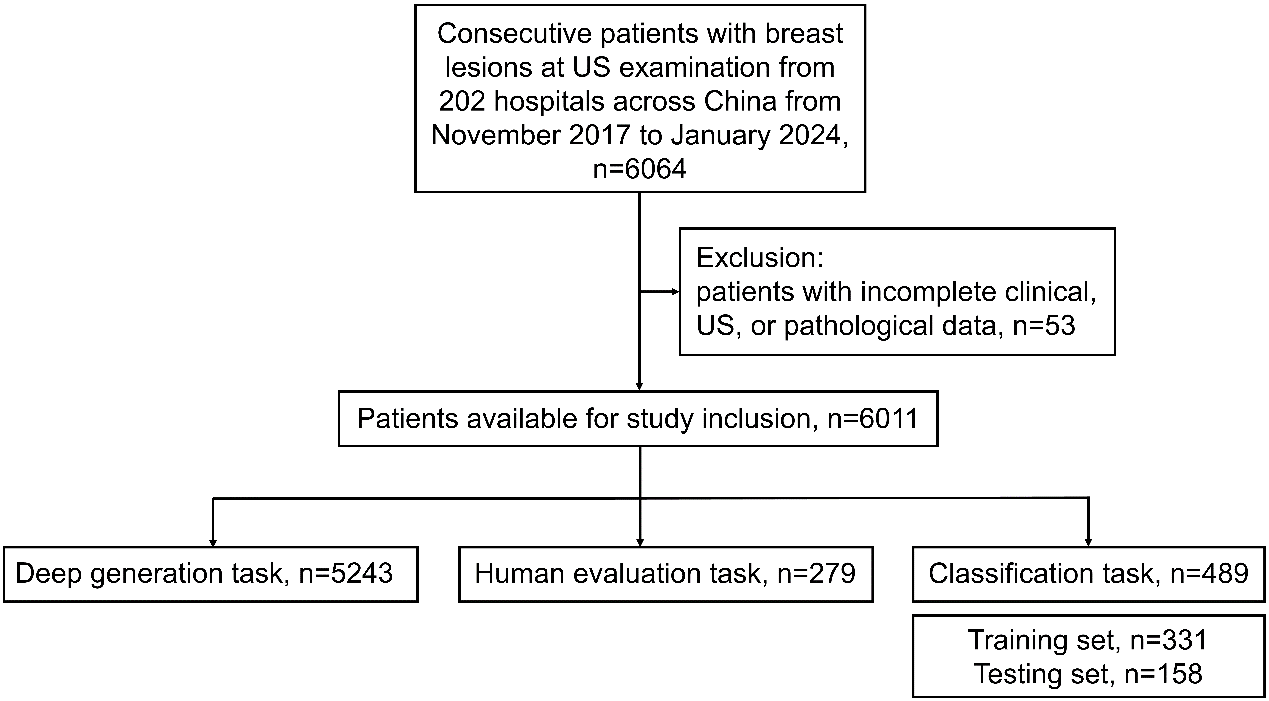


**Figure S1**: Flowchart of inclusion and exclusion criteria for patients.


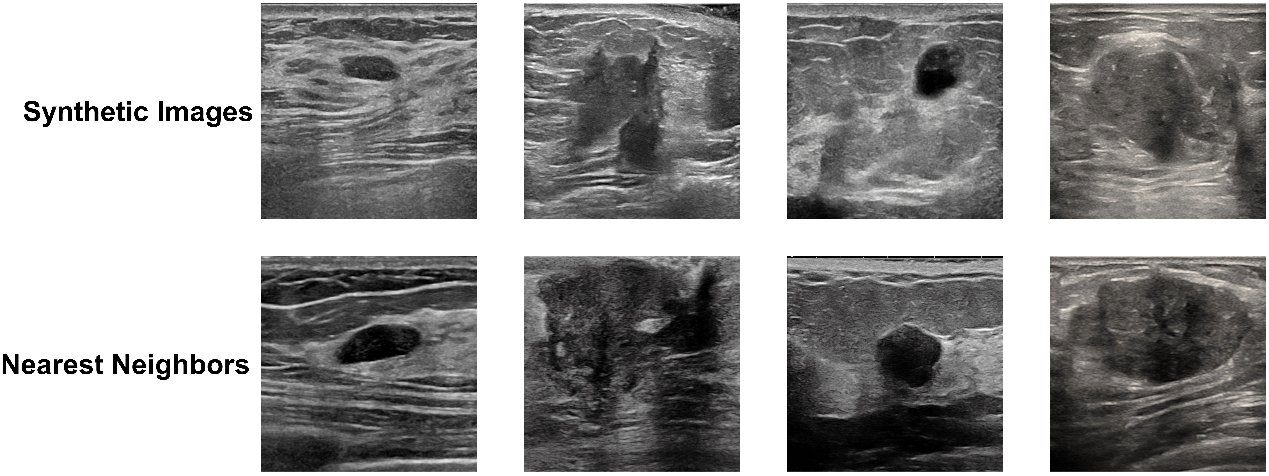


**Figure S2**: Synthetic breast US images with their nearest neighbors. The first row displays randomly selected synthetic breast US images generated by CoLDiT, while the second row shows the nearest neighbor from the training set for each synthetic image. The images are vertically aligned in pairs, with nearest neighbors identified using cosine similarity.
