# Supplementary figures and images for: Synthetic Breast Ultrasound Images: A Study to Overcome Medical Data Sharing Barriers
Source: Research (Wash D C). 2024 Dec 3;7:0532. doi: 10.34133/research.0532 (PMC11612121; doi:10.34133/research.0532)

**Synthetic Images**

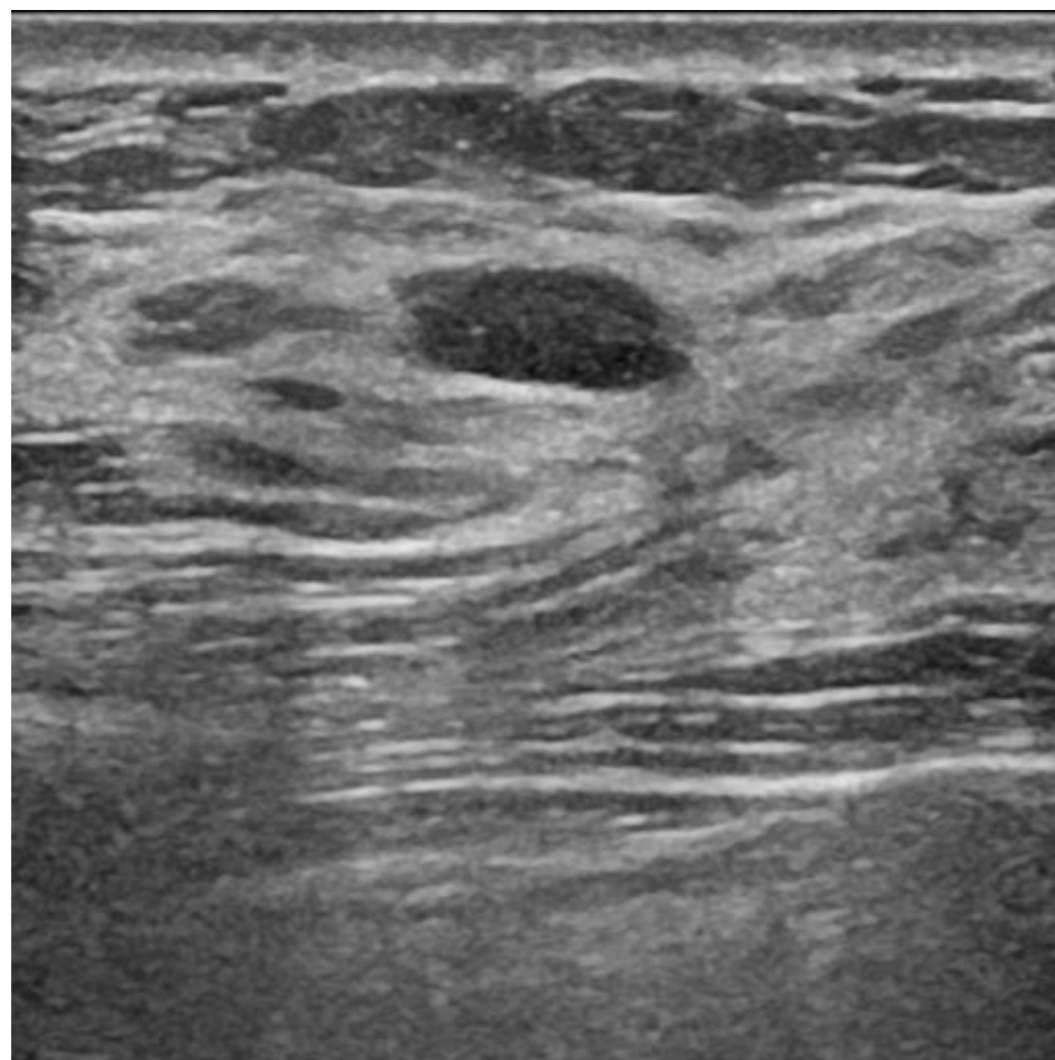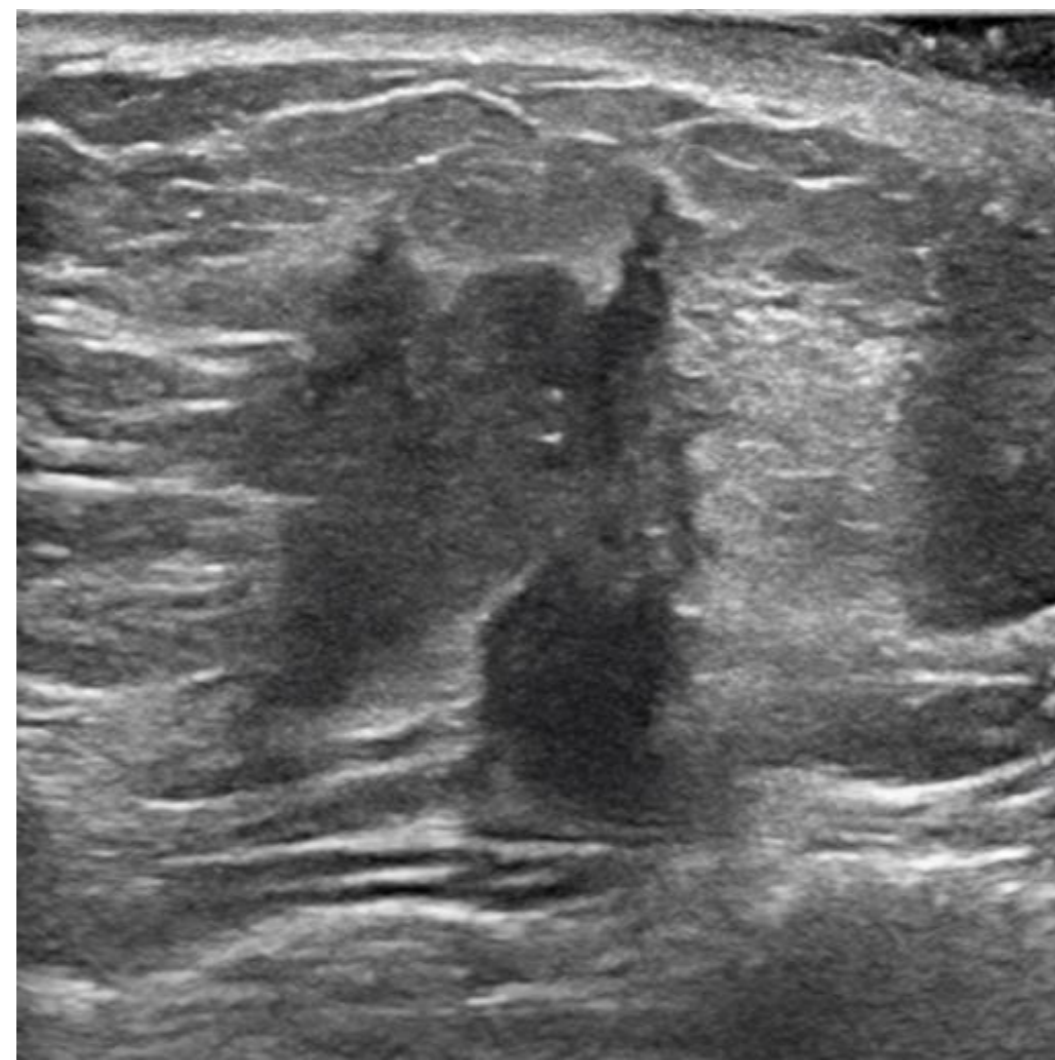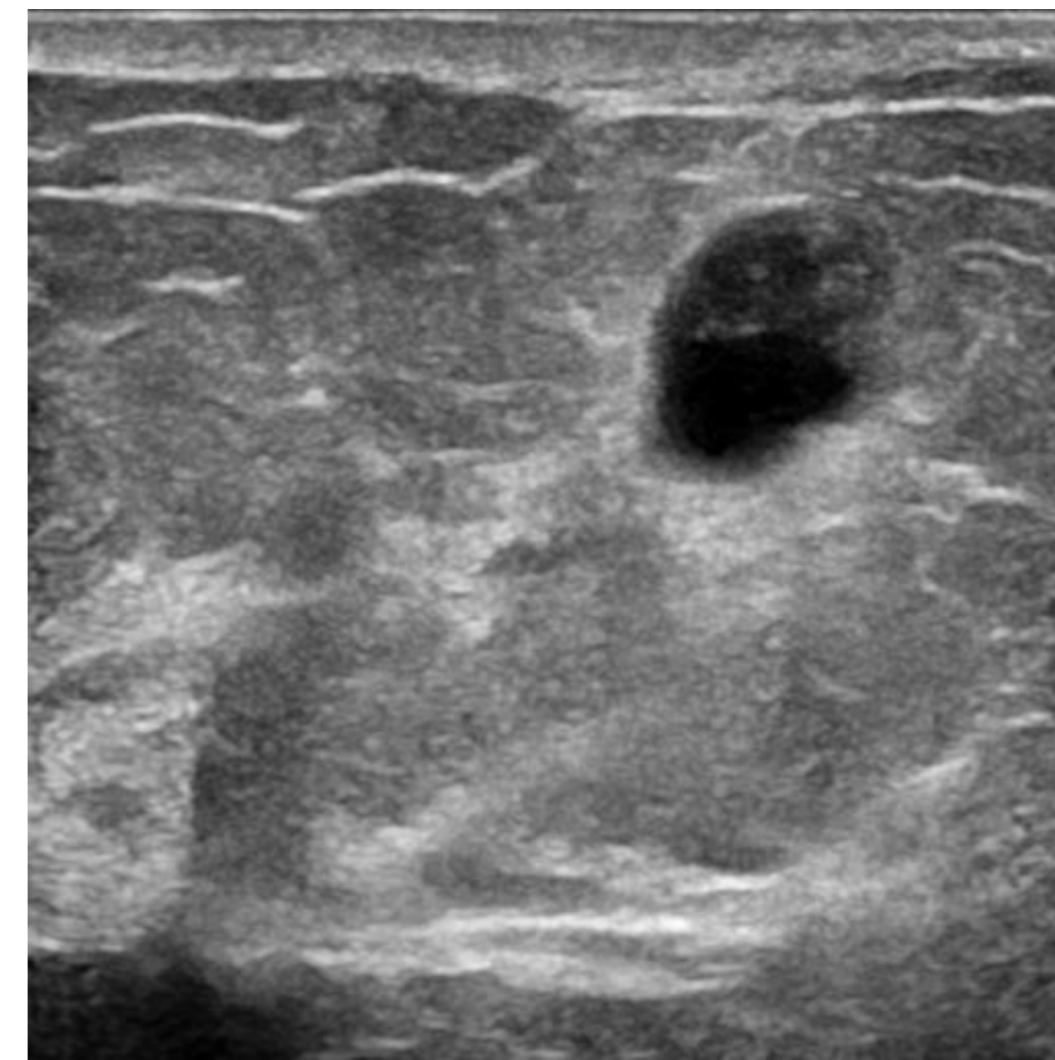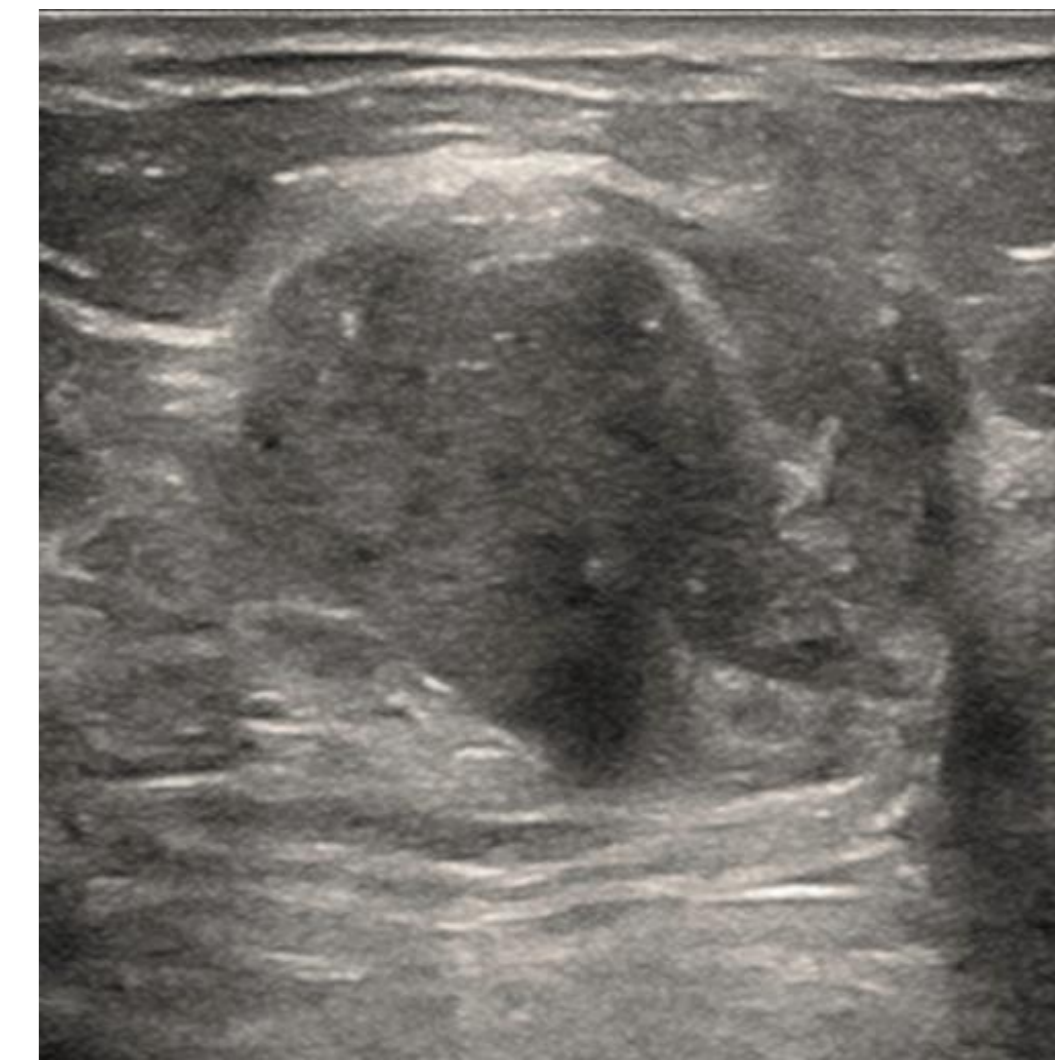

**Nearest Neighbors**

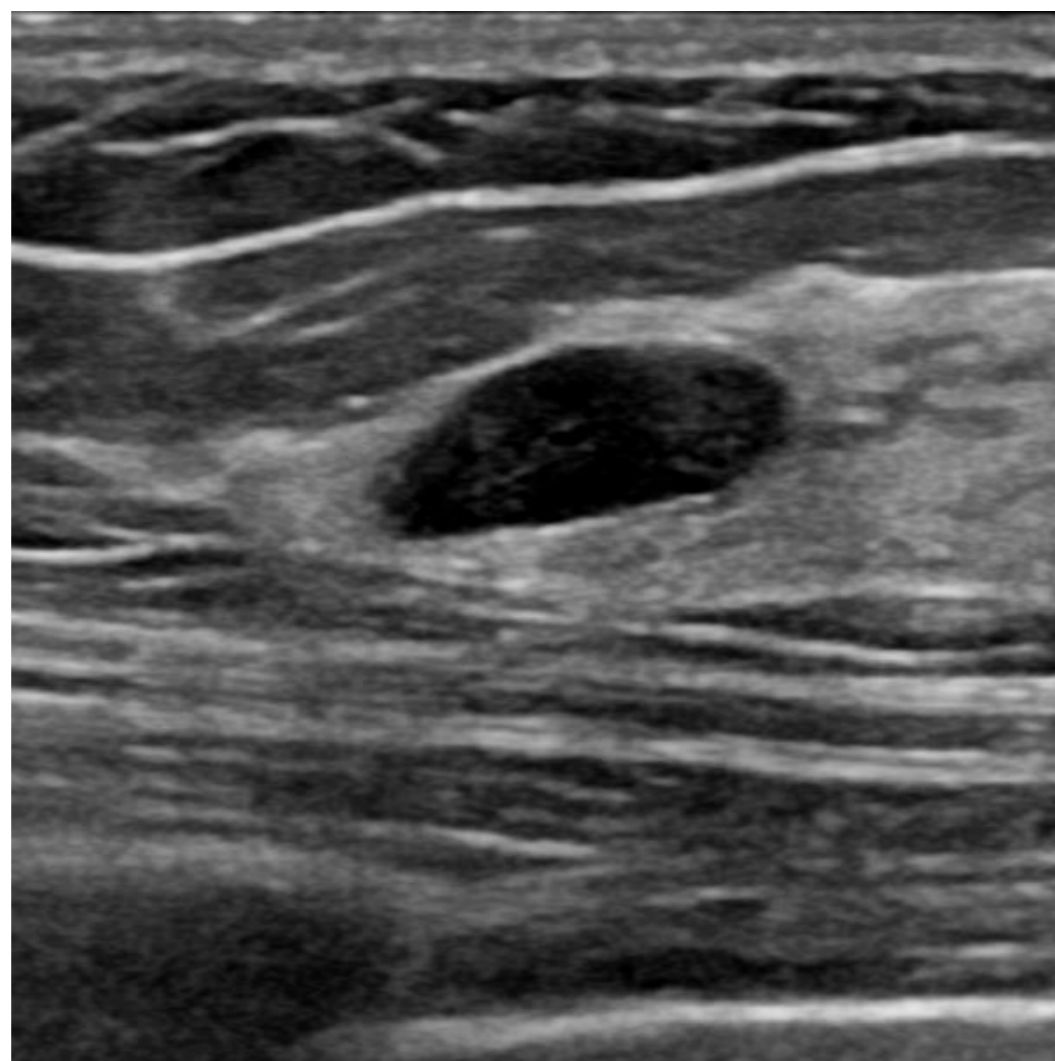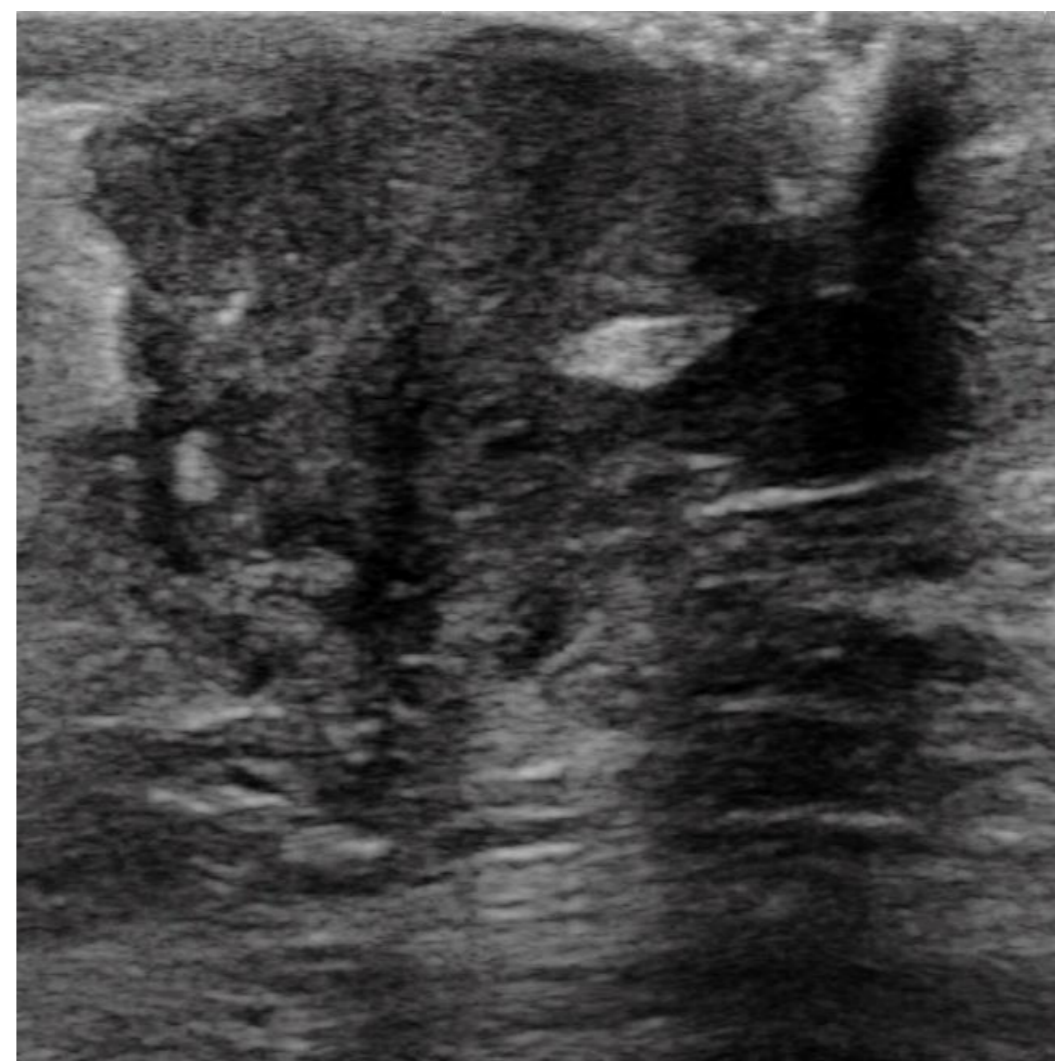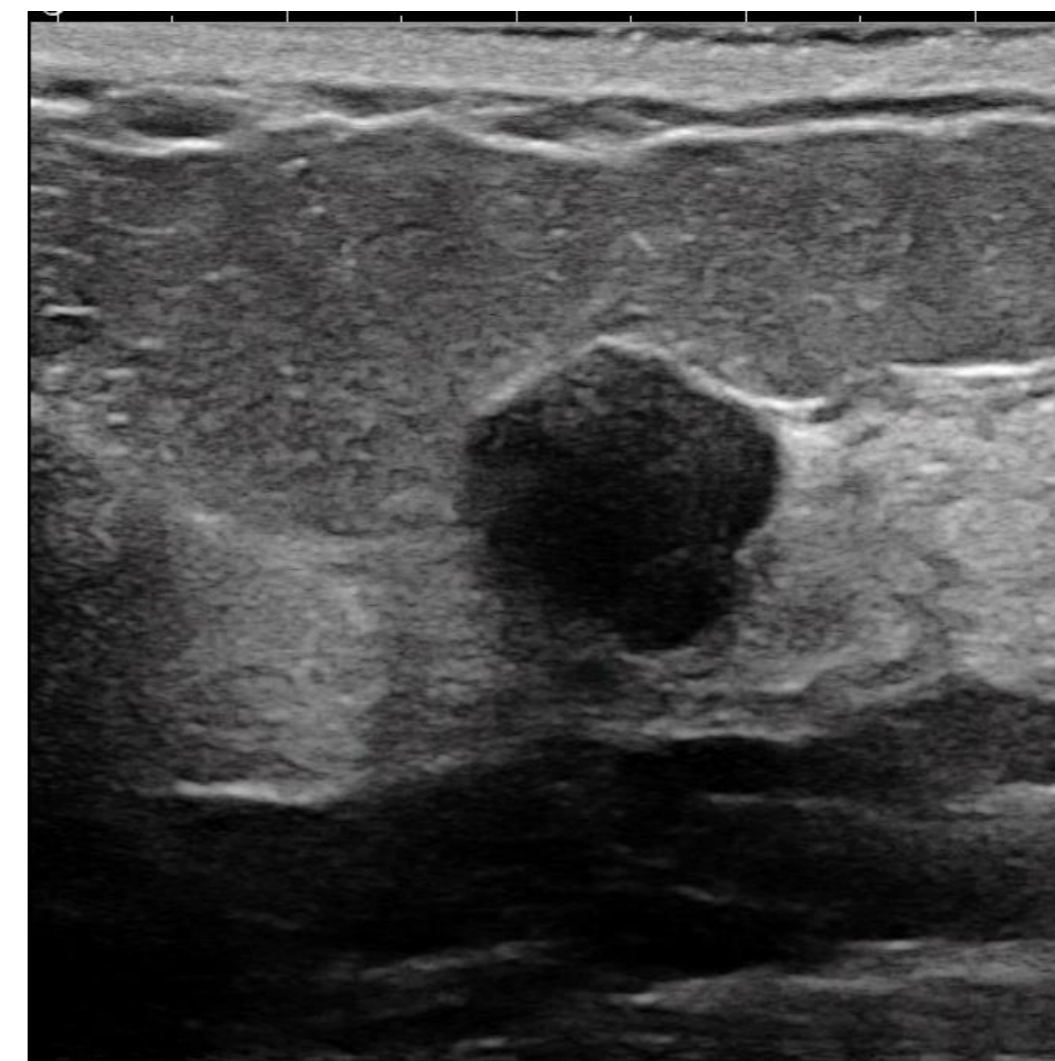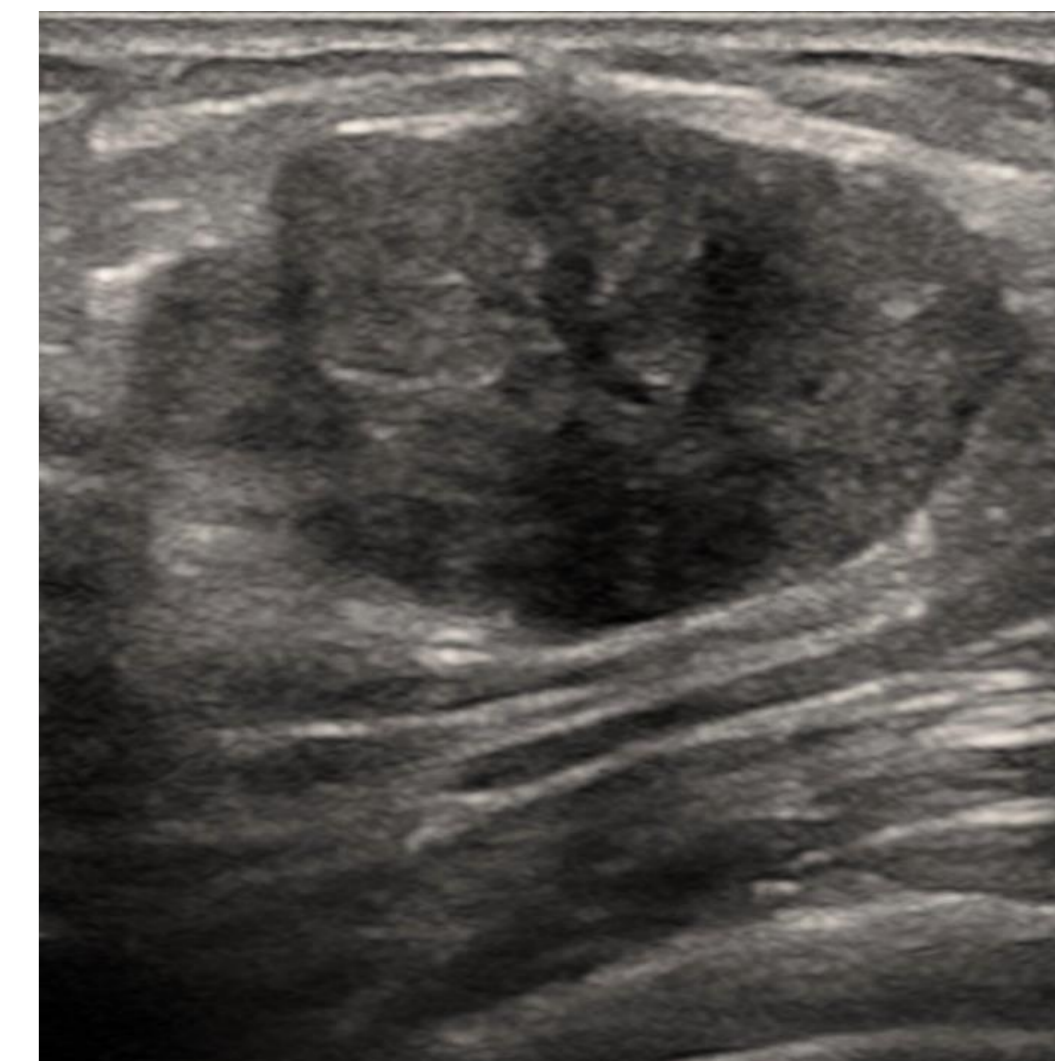

Supplement: Supplementary 1 — Appendix S1 Figs. S1 and S2 Tables S1 to S3 [file research.0532.f1.zip › figS2.pdf]
